# Pretreatment with *Salvadora persica* L. (Miswak) aqueous extract alleviates paracetamol-induced hepatotoxicity, nephrotoxicity, and hematological toxicity in male mice

**DOI:** 10.14202/vetworld.2021.589-594

**Published:** 2021-03-08

**Authors:** Mohd Alaraj, Tolgahan Acar, Irena Kosinska, Bahaa Al-Trad, Ammar M. Almaaytah, Mohamed J. Saadh, Mohammed A. Qumani, Shahid M. Syed, Khalil I. Altaif, Hossain Ashfaque

**Affiliations:** 1 Department of Pharmacy, Faculty of Pharmacy, Middle East University, Jordan; 2 Department of Physical Therapy, College of Applied Medical Sciences, University of Hail, Saudi Arabia; 3 Department of Social Medicine and Public Health, Medical University of Warsaw, Poland; 4 Department of Biological Sciences, Yarmouk University, Irbid, Jordan; 5 Department of Pharmaceutical Technology, Faculty of Pharmacy, Jordan University of Science and Technology, Irbid, Jordan; 6 Department of Pharmacology, College of Medicine, University of Hail, Saudi Arabia; 7 Department of Medical Microbiology, Ras Al Khaimah Medical and Health Sciences University, Ras Al Khaimah, UAE

**Keywords:** hematology, kidney, liver, mice, paracetamol, *Salvadora persica* L

## Abstract

**Background and Aim:**

Paracetamol (PCM) ingestion is one of the most frequent global causes of toxicity. *Salvadora persica* L. is a plant that among many other effects exhibits potent antioxidant, anti-inflammatory, antimicrobial, and anticancer effects. In this study, we investigated the possible protective effect of *S. persica* aqueous extract in the PCM overdose-induced liver and kidney injury and hematological changes in a mice model.

**Materials and Methods:**

Mice were given PCM with and without *S. persica* pretreatment. Blood cell counts and liver and kidney function biomarkers were measured. Liver and kidney samples were histologically examined.

**Results:**

A single overdose of PCM caused significant elevations of alanine and aspartate transaminases, alkaline phosphate, bilirubin, urea, uric acid, and creatinine compared with the control group. In addition, PCM toxicity significantly lowered red blood cell count but insignificantly increased both white blood cell and platelet counts in comparison to the control mice. Pretreatment with *S. persica* significantly prevented PCM-induced changes in hepatic and renal biomarkers. *S. persica* also caused marked reversal of hematological changes. Histologically, the liver and kidney showed inflammation and necrosis after PCM treatment, which were significantly reduced in mice pretreated with *S. persica*.

**Conclusion:**

Taken together, *S. persica* significantly inhibited PCM-induced renal, hepatic, and hematological toxicity, pointing to its possible use in the treatment of liver and renal disorders.

## Introduction

Paracetamol (PCM) is the safest and most widely consumed minor analgesic and antipyretic drug across the world [1]. However, overdose with PCM can induce potentially deadly hepatotoxicity [2,3]. PCM toxicity is a leading cause of liver malfunction in developed countries [4], but recent data show that PCM overdose also causes renal failure more frequently than previously reported and this can occur even without liver damage [5]. It has been well established that hepatotoxicity of PCM is caused by the toxic metabolite N acetyl-p-benzoquinone imine (NAPQI), which is conjugated with intracellular glutathione. PCM overdose leads to high levels of NAPQI which, after depleting the glutathione, causes oxidative stress and consequently liver damage [3]. In PCM-induced nephrotoxicity, many studies implicate oxidative stress, in addition to prostaglandin and N-deacetylase enzyme [5-7].

*Salvadora persica*, the desert plant family of *Salvadoraceae* has a long history as a medicinal herb and is commonly used as a tooth-cleaning stick in many African and Middle Eastern countries [8]. It contains carbohydrates, flavonoids, volatile oils, alkaloids, steroids, terpenoids, and saponin [9,10]. Sticks from *S. persica* also contain a substantial amount of silica [11], three lignin glycosides [12], and minor components such as volatile oils and flavonoids [13]. Toxicity studies using laboratory animals show that the aqueous extract of *S. persica* is quite safe [14]. *S. persica* exhibits antimicrobial, anti-inflammatory, antipyretic, antioxidant, anticancer, and other activities [15-17]. In the context of gentamicin-induced nephrotoxicity and hepatotoxicity, *S. persica* supplementation attenuates oxidative stress and prevents the development of gentamicin-induced acute renal and liver toxicity [18].

Although there are several known pharmacological effects of *S. persica*, a protective effect of *S. persica* against PCM-evoked hepatotoxicity and nephrotoxicity remains unknown. In this study, the beneficial properties of the aqueous extract of *S. persica* on the liver and kidney and hematological indices of PCM-intoxicated male mice were evaluated.

## Materials and Methods

### Ethical approval

The protocol of this study was approved by the ethics board of animal experiments, University of Hail, Saudi Arabia. All experimental procedures were conducted according to the guidelines set by the World Health Organization (Geneva, Switzerland).

### Study period and location

This study was conducted from March 2018 to August 2018 at the University of Hail, Hail, Saudi Arabia.

### Animals

A total of 32 male Swiss albino mice, 5 weeks old and weighing 20-25 g, were obtained from the Faculty of Science Animal House, University of Hail. Mice were kept in stainless-steel cages maintained in the animal house facility with 12-h light/dark cycles at 23ºC±2°C with a relative humidity of 50-60%. They were fed a standard commercial mouse diet and were acclimatized and routinely observed under housing conditions for 1 week before use in the experiment.

### Chemicals

Distilled water, 10% formalin, hematoxylin, eosin, and PCM were obtained from Merck (Germany).

### Preparation of *S. persica* L. (Miswak) extraction

Dried stems of *S. persica* were purchased from the local market and milled to powder. The powder (5 g) was boiled for 30 min in 100 mL distilled water, filtered, and lyophilized. The lyophilized powder was given to mice at dose of 500 mg per kg body weight per day in an aqueous vehicle, in a volume of 0.4 mL/10 g body weight.

### Experiment design

Mice were grouped into four groups of eight animals each, as follows: Control group mice received distilled water (0.4 mL/10 g body weight per day) orally for 15 days and then an intraperitoneal injection (i.p.) of distilled water on day 16. PCM group mice received distilled water (0.4 mL/10 g body weight per day) for 15 days and then a single 500 mg/kg i.p. dose of PCM on day 16 [19]. *S*. *persica* group mice were administered only oral *S. persica*, 500 mg/kg per day [18] for 15 days. *S. persica* and PCM group mice received oral *S. persica*, 500 mg/kg per day for 15 days and on the 16^th^ day received 500 mg/kg i.p. PCM.

Blood samples were collected 2.5 h after PCM administration from the retroorbital plexus of anesthetized mice in all groups, using nonheparinized ­hematocrit capillaries [19]. Plasma was separated by centrifugation at 3000 rpm for 10 min before biochemical analysis.

### Blood analysis

In plasma samples, urea was quantified using an enzymatic colorimetric method (ureas), uric acid using an enzymatic colorimetric method (uricase), bilirubin using the Jendrassik–Grof method, and creatinine using the kinetic alkaline picrate method (Jaffe). Alanine transaminase (ALT), aspartate transaminase (AST), and alkaline phosphate (ALP) levels were quantified using modified International Federation of Clinical Chemistry kinetic methods, and activity of the enzymes was measured using commercial kits according to the manufacturer’s instructions (Arcomex, Jordan) [20]. Various hematological parameters, such as red blood cell (RBC), white blood cell (WBC), and platelet counts, were estimated.

### Histopathological studies

After blood collection, the mice were sacrificed and their livers and kidneys dissected out and washed with phosphate buffer (pH 7.4) to remove clots and blood stains. The tissue samples were then fixed in 10% formalin and embedded in molten paraffin wax. Tissue sections were cut with a microtome. Deparaffinized sections were stained with hematoxylin and eosin and histopathological changes analyzed using a microscope.

### Statistical analysis

SPSS version 18.0 (Chicago, IL) was used for statistical analyses. Data were described as mean ± SEM. Analysis between two groups was performed using Student’s t-test. Normality was assessed using the Kolmogorov–Smirnov test. Continuous variables were described as mean±standard deviation. Differences between groups were obtained using one-way analysis of variance tests with Tukey–Kramer HSD for *post hoc* analysis. Differences were considered significant at p<0.05.

## Results

### Effects of PCM and *S. persica* on liver and kidney function

Compared with the control group treated with distilled water, mice treated with single dose of PCM (PCM group) had significantly increased (p<0.001) activity of the enzymes AST, ALT, and ALP and concentration of bilirubin, creatinine, urea, and uric acid (Table-1). However, there were no significant differences in any of these biochemical parameters between animals treated only with *S. persica* (*S. persica* group) and control animals. Mice administered *S. persica* (500 mg/kg body weight per day for 15 days) and then PCM (*S. persica* and PCM group) ­demonstrated marked protection against PCM-induced hepatocellular damage, as revealed by significant reduction (p<0.01) in the elevated serum levels of AST, ALT, ALP, creatinine, and uric acid seen in mice that received PCM only (PCM group) (Table-1). Likewise, pretreatment with the *S. persica* extract (*S. persica* and PCM group) had significantly (p<0.05) lower serum levels of bilirubin and urea than the elevated levels in the PCM group (treated with PCM only) (Table-1).

**Table-1:** Effect of *Salvadora persica* on liver and kidney linked biomarkers of PCM-intoxicated male mice.

Biochemical marker	Control group	PCM group	S. group	S. and PCM group
AST (IU/L)	23.14±3.11	54.89±1.98***	25.11±4.10	34.17±1.76**
ALT (IU/L)	31.47±1.12	61.79±3.37***	30.45±1.65	40.32±1.89**
ALP (IU/L)	187.87±11.76	305.12±13.12***	189.11±10.21	241.13±13.11**
Bilirubin (mg/dL)	0.38±0.09	2.56±0.21***	0.41±0.11	1.44±0.17*
Creatinine (mg/dL)	0.58±0.039	2.41±0.31***	0.57±0.047	1.54±0.16**
Urea (mg/dL)	18.94±1.12	53.13±7.14***	19.89±1.58	40.12±3.19*
Uric acid (mg/dL)	0.59±0.083	1.84±0.13***	0.61±0.093	0.87±0.14**

* p≤0.05,

** p≤0.01,

*** p≤0.001.

Values are expressed as mean±SEM (n=7), Group 2 (PCM group) was compared with Group 1 (vehicle control). Group 3 (*Salvadora persica* group) was compared with Group 1 (vehicle control). Group 4 (*Salvadora persica* and PCM) was compared with Group 2 (PCM group). AST=Aspartate aminotransferase, ALT=Alanine transaminase, ALP=Alkaline phosphatase, ALP=Alkaline phosphate, PCM=Paracetamol

### Effect of *S. persica* on hematological parameters in PCM-intoxicated male mice

PCM overdose significantly lowered RBC count but caused statistically insignificant elevation of WBC and platelet count in comparison to the control animals. Pretreatment with *S. persica* reversed these alterations, significantly decreasing the toxicity of PCM (Table-2).

**Table-2:** Effect of *Salvadora persica* on blood cells count of paracetamol - intoxicated male Mice.

Biochemical marker	Control group	Paracetamol group	S. group	S. and Paracetamol group
White blood cell (10≥/mm≥)	6.53±0.23	8.89±0.98	6.67±0.31	6.41±0.26
Red blood cell (10◻/mm^3^)	8.57±0.752	5.19±0.37*	8.45±0.65	8.32±0.89α
Platelets (mm^3^)	636±33.3	712±23.12	641±10.21	631±27.11

* p<0.05 as compared to the control group, ^α^p<0.05 in comparison to paracetamol treated group

### Effect of *S. persica* on PCM-induced histopathological changes

**Figure-1:**
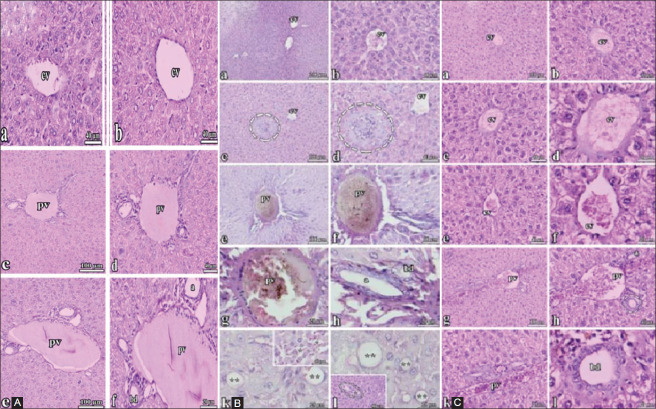
(A) Control (Liver): General structure of liver in this group was normal. The wall of central vein (cv) was well preserved in the classic hepatic lobule, the structures in the portal triads were observed in normal, portal vein (pv), a branch of pv ; (a), a hepatic artery; (bd), bile duct. The sinusoids between cords of hepatic cells were fine. Hematoxylin and eosin staining. (B) Paracetamol (Liver): General structure of liver in this group was abnormal, since enlarged sinusoids (*) were commonly seen between hepatic cords. The wall of cv was in normal and seen in the center of the classic hepatic lobule. The structures in the portal triads were abnormal appearance, its wall was thicker and other structures in the portal triad were not seen clearly. An unknown structure that was a cluster of cells near the cv was observed (c, d). Lipid stored cells were commonly seen (**). (pv) a branch of pv; (a) a hepatic artery; (bd) bile duct. Hematoxylin and eosin staining. (C) *Salvadora persica* + PMC (Liver): General structure of liver in this group was normal. The wall of cv was clearly seen in the center of the classic hepatic lobule. The sinusoids between the cords of hepatic cells were fine. The structures in the portal triads were in normal appearance, (pv) a branch of pv; (a) a hepatic artery; (bd) bile duct. Hematoxylin and eosin staining.

**Figure-2:**
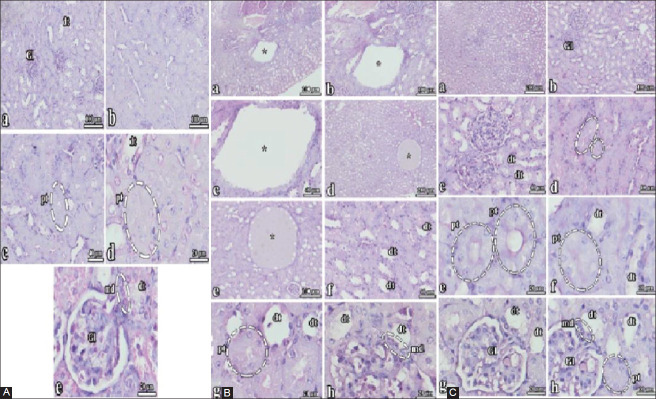
(A) Kidney-control: General structure of the kidney in this group was normal. The borders of proximal and distal tubules as well as the glomerulus were clearly observed. The distal tubules that close vicinity of the glomerulus have macula densa in its wall (md). (Gl), glomerulus; (dt), distal tubule; (pt), proximal tubule. Hematoxylin and eosin staining. (B) Paracetamol (PCM) (kidney): General structure of the kidney in this group at low magnification (a) was not seen normal. At the high magnification it was not only observed many dilated spaces but also some of the kidney cysts (*) (a, b, c, d, e). The dilated tubules were common in the kidney. Glomerulus of this group has a hemorrhagic structure that might result in dilated capillaries. These capillaries were filled with blood cells, outside of glomerulus in the kidney hemorrhagic structures could also be seen (a, b). In some regions, the border of macula densa in the distal tubule would be seen. Even some well-preserved proximal tubules were observed, but the distal tubules in the kidney were generally dilated. The distal tubule that was a close vicinity of the glomerulus has a macula densa (md) in its wall. (Gl), glomerulus; (dt), distal tubule; (pt), proximal tubule. (C) *Salvadora persica* + PCM (kidney): General structure of the kidney in this group was look fine. Since a well-preserved tissue, the borders of proximal and distal tubules as well as the glomerulus were clearly observed in the kidney at low and high magnification. The distal tubules that were the close vicinity of the glomerulus have a macula densa (md) in its wall. The borders of glomerulus were clear. (Gl), glomerulus; (dt), distal tubule; (pt), proximal tubule. Hematoxylin and eosin staining.

In the control group, liver and kidney samples were morphologically normal (Figures-1A, 2B). The liver samples from mice that received PCM showed apparent cellular injury, with a congested central vein, shrunken nuclei, and enlarged sinusoids (Figure-1B). The liver samples from mice pretreated with *S. persica* extract before PCM had normal architecture of the liver tissues, showing that the liver cells, central vein, and portal vein were protected from PCM toxicity (Figure-1C). The kidney samples of the PCM group showed dilated tubules, hemorrhagic glomeruli, and other alterations (Figure-2B). However, the kidneys of the *S. persica*-treated group were intact after PCM administration (Figure-2C).

## Discussion

Overdose or long-term use of certain medications is clearly linked with liver injury, kidney injury, or both. High dosage or chronic use of PCM is a very famous example of this pathology; this analgesic is known as a life-threatening agent due to its both marked hepatic and renal impairment [21]. PCM also induces substantial hematological changes in animals [22]. Consequently, medicinal plants capable of protecting the liver, kidney, and blood from PCM-induced toxicity are urgently needed.

Hepatic cells manufacture the enzymes ALT, AST, and ALP, and elevated circulating levels of these enzymes indicate injured hepatic cells [23]. Similarly, increases in serum urea, uric acid, and creatinine are key indicators of kidney failure and tissue injury [24]. Oxidative stress is an essential mechanism of PCM toxicity. Specifically, accumulation of the toxic metabolite of PCM and NAPQI is responsible for acute centrilobular hepatic necrosis that causes cell dysfunction and destruction of the hepatocellular membrane [25,26]. Similarly, throughout the process of drug excretion, NAPQI in high concentrations is able to bind irreversibly with numerous renal cellular proteins, causing mitochondrial dysfunction and inducing renal damage [27,28]. This study showed that in PCM-treated mice, liver-related parameters, that is, ALT, AST, and ALP serum levels, significantly increased and microsections of liver revealed necrosis and infiltration of inflammatory cells. Comparable results were previously seen in a study carried out by Karabacak *et al*. [29]. In our experimental model, pretreatment with *S. persica* extract protected against PCM-induced hepatotoxicity. This was demonstrated by reduction of the elevated liver function markers and intact histology of liver in mice treated with *S. persica* before PCM.

Similarly, this study demonstrated an obvious increase of all measured kidney function markers in PCM-treated animals and marked histopathological alterations of the kidney, confirming that PCM-induced kidney injury. This rise in kidney enzymes following high-dose PCM was similar to the findings of other studies [5,20]. However, pretreatment with *S. persica* significantly improved the kidney enzyme profile after PCM administration, indicating that *S. persica* has a nephroprotective effect in the context of PCM-induced nephron toxicity; and PCM-intoxicated mice pretreated with *S. persica* showed a significant reduction in the serum concentrations of urea, uric acid, and creatinine. Kidney microsections from mice pretreated with *S*. *persica* extract before PCM also showed marked reduction of necrosis and kidney tissue degeneration. *S. persica* has previously been found to have comparable protective properties against gentamicin-induced renal and liver toxicity [18]. These hepatoprotective and nephroprotective properties are probably due to the antioxidant and anti-inflammatory actions of the plant extract constituents, such as flavonoids, alkaloids, glycosides, steroids, carbohydrates, tannins, and saponins [10].

This study also showed that PCM overdose caused a significant (p<0.05) reduction in RBC count but only a slight increase in both WBC and platelet counts compared to control animals. These findings are comparable with earlier results from a study conducted by Menyiy *et al*. [22]. Destruction of RBCs is caused by lipid peroxidation, membrane protein cross-linking, and fragmentation triggered by free radicals [28]. Pretreatment with the *S. persica* extract significantly attenuated these hematological alterations in PCM-intoxicated mice, probably due to the antioxidative and anti-inflammatory effects of this plant.

## Conclusion

Taken together, these data show that oral administration of *S. persica* extract (Miswak) significantly protects against the hepatic, renal, and hematological toxicity induced by PCM overdose. This suggests that *S. persica* may have benefits in the prevention of liver and renal disorders. The observed protective capacity of Miswak is likely due to its anti-inflammatory and antioxidant properties.

## Authors’ Contributions

MA, MJS, MAQ, and KIA designed the study, wrote the manuscript, and participated in conducting the experiment. IK, BA, and SMS performed the *in vivo* experiment and collected the samples. TA, AMA, and SMS performed the histological investigations. HA and AMA processed and analyzed the data. All authors read and approved the final manuscript.
